# Postoperative contact dermatitis caused by skin adhesives used in orthopedic surgery: Incidence, characteristics, and difference from surgical site infection

**DOI:** 10.1097/MD.0000000000026053

**Published:** 2021-05-21

**Authors:** Sang Pil So, Jae Youn Yoon, Ji Wan Kim

**Affiliations:** a Department of Orthopedic Surgery, Asan Medical Center, University of Ulsan, College of Medicine, Seoul, Republic of Korea.; b Department of Orthopaedic Surgery, Dongguk University Ilsan Hospital, Goyang, Republic of Korea.

**Keywords:** contact dermatitis, skin adhesive, surgical site infection

## Abstract

Skin adhesives are used to close clean surgical wounds. We aimed to investigate the incidence of skin adhesive-related contact dermatitis and the characteristics that differentiate it from a surgical site infection.

We retrospectively analyzed patients whose surgical wound was closed using a liquid skin adhesive (Dermabond Prineo skin closure system, Ethicon, NJ) by a single surgeon between March 2018 and June 2020. Medical records were reviewed to evaluate complications indicating contact dermatitis, including wound infections and hematomas.

We included 143 patients (men, 59; women, 84; mean age, 60.8 years). No patient had an early surgical site infection or wound dehiscence, but 4 (2.8%) developed postoperative contact dermatitis (week 7, 1; week 4, 2; day 9, 1). Manifestations included eczema and pruritus, without local heat or wound discharge. All cases resolved without complications, including infection.

Contact dermatitis occurred in 2.8% of patients who received liquid skin adhesive, and the symptoms differed from those of surgical site infection. Patients should be informed about the risk of contact dermatitis before applying a liquid skin adhesive.

## 1 Introduction

Skin adhesives are used to close the skin in clean surgical procedures. Compared with previous closure methods, this technique is pain-free, requires no suture removal, and has similar cosmetic outcomes.^[1,2]^ Skin adhesives are easily and swiftly applied,^[3]^ seal the wound and increase the strength of the wound closure,^[4,5]^ and protect against microorganisms.^[6]^ However, complications of skin adhesives, including skin defects, infections, ulcers, and allergic contact dermatitis caused by a type IV (delayed) hypersensitivity reaction, have been reported.^[5,7,8]^

Contact dermatitis can be confused with surgical site infection (SSI) and result in a secondary SSI due to the skin's loss of function as a barrier to infection.^[9,10]^ As skin adhesives are now widely used for surgical wound closure, research is needed to accurately characterize the contact dermatitis that can result from their use. We aimed to evaluate the incidence, characteristics, and treatment outcomes of contact dermatitis caused by using skin adhesives and differentiate it from SSI.

## 2 Materials and methods

This study was conducted with the approval of our institution's institutional review board. We retrospectively analyzed patients whose surgical incisions were closed using a liquid adhesive skin closure system (Dermabond Prineo, skin closure system, Ethicon Inc., Somerville, NJ) containing 2-octyl acrylate, from March 2018 to June 2020. One surgeon performed all the procedures. The inclusion criteria were: a skin adhesive was the only skin closure material, patient age ≥20 years, and follow-up ≥6 months postoperatively. The exclusion criteria were: surgery due to infection, history of chronic skin disease, no medical records.

When using the skin adhesive during surgery, the subcutaneous layer was sutured using vicryl sutures. Liquid adhesive and mesh were applied to the skin layer. The mesh was removed 2 weeks postoperatively. Surgical drains were inserted at the sites where the soft tissue layer was thin, including the distal tibia.

We reviewed the medical records to identify patients who received the skin adhesive for skin closure; collected the demographic data; and recorded the occurrence of postoperative wound infections, skin re-closures, and hematomas. Skin adhesive-related allergic contact dermatitis was diagnosed by dermatologists based on the patient's clinical manifestations, including the characteristics and site of the skin lesion, without further patch tests. In the dermatology clinic, topical steroids were applied to the wound and oral antihistamines were prescribed to relieve pruritis around the wound. The incidence of contact dermatitis due to the use of skin adhesives, and its symptoms, signs, treatment, and prognosis were evaluated. Furthermore, we identified the characteristics of contact dermatitis that differentiate this disease from SSI.

## 3 Results

We enrolled 143 patients (men, 59; women, 84; mean age, 60.8 years [range, 23–93 years]). All patients underwent lower extremity surgery (hip arthroplasty, 114 cases, Table 1). There were no cases of early SSI or wound dehiscence. However, 4 patients were diagnosed with contact dermatitis (2.8%).

**Table 1:** Procedures.

Operation	Number
Total hip replacement arthroplasty	91
Bipolar hemiarthroplasty	23
Intramedullary nailing for hip fracture	4
Open reduction and internal fixation	13
Revision hip arthroplasty	2
Implant removal	9
Femoral osteotomy	1
Total	143

### 3.1 Clinical manifestations of contact dermatitis

All the 4 patients had eczema and pruritis around the surgical wound, without local heat or wound discharge. The average postoperative time to the diagnosis of contact dermatitis was 3.9 weeks (range, 9 days to 7 weeks). After the diagnosis of contact dermatitis, a mesh applied at the surgical site was immediately removed, when it was not removed previously. All the patients’ conditions resolved without complications, including infection, after short-term treatment with an antihistamine and topical steroid ointment.

#### 3.1.1 Case 1

**Figure 1:**
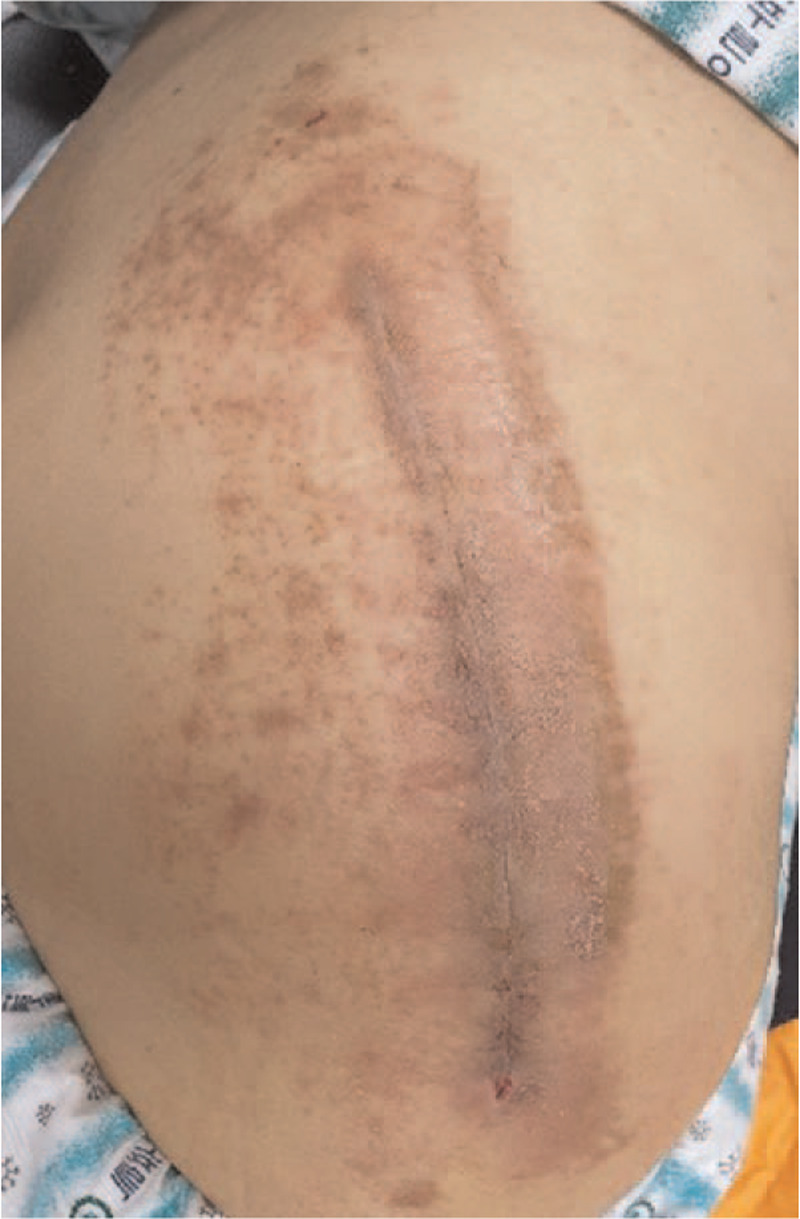
A 71-year-old woman presented with an itchy eczematous lesion around the surgical wound on postoperative day 9.

A 71-year-old woman, discharged after total hip replacement arthroplasty, returned to the hospital on postoperative day 9 with a 3-day history of itchy eczematous lesion around the surgical wound (Fig. 1). The patient was afebrile, and there was no warmth, redness, or tenderness on wound examination. A blood test at that time showed the following: white blood cell (WBC) count, 5100/μL (normal 4000–10,000/μL); erythrocyte sedimentation rate (ESR), 84 mm/h (normal 0–20 mm/h); and C-reactive protein (CRP), 4.43 mg/dL (normal 0–0.6 mg/dL). The patient's inflammatory markers were mildly elevated compared with the blood test results 5 days earlier: WBC, 3600/μL; ESR, 30 mm/h; and CRP, 2.46 mg/dL. Because SSI could not be ruled out, the patient received cefazolin, 2 g, intravenously. The next day, the patient was diagnosed with contact dermatitis at the dermatology clinic and treated with local steroid ointment without additional antibiotics. Also, the mesh applied at the surgical wound was removed immediately. The skin lesion resolved after 1 week of treatment.

#### 3.1.2 Case 2

**Figure 2:**
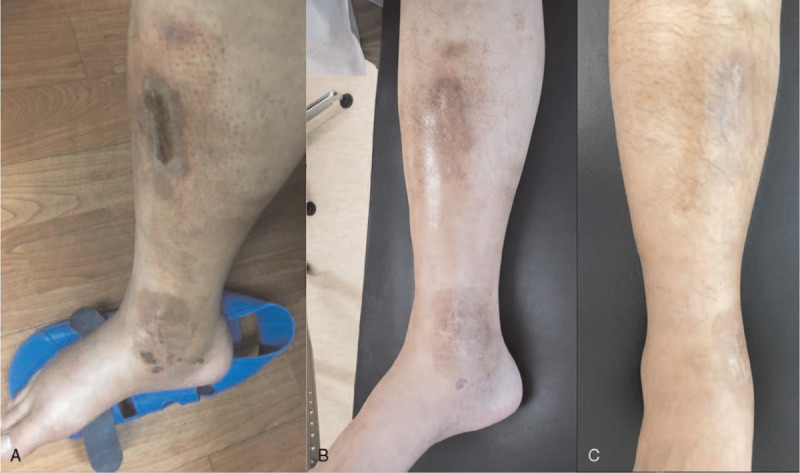
A 30-year-old man treated with minimally invasive plate osteosynthesis for a distal tibio-fibular fracture: (A) an itchy erythemato-edematous vesicular lesion at the 4-week postoperative follow-up, (B) a photograph at the 2-week post-treatment follow-up, (C) after 4 weeks of treatment, the contact dermatitis resolved without complications.

A 30-year-old man developed an itchy erythemato-edematous vesicular lesion around his surgical wound on the fourth postoperative week (Fig. 2A). A routine blood test at the first outpatient clinic follow-up (4 weeks postoperatively) showed: WBC, 9500/μL; ESR, 12 mm/h; and CRP, 0.1 mg/dL. The patient was referred to the dermatologic clinic where he was diagnosed with contact dermatitis and skin infection, and prescribed oral antibiotics for 3 days and a local steroid ointment. The patient's pruritis resolved 2 weeks later (Fig. 2B), and the skin lesions resolved after 4 weeks of treatment (Fig. 2C).

#### 3.1.3 Case 3

**Figure 3:**
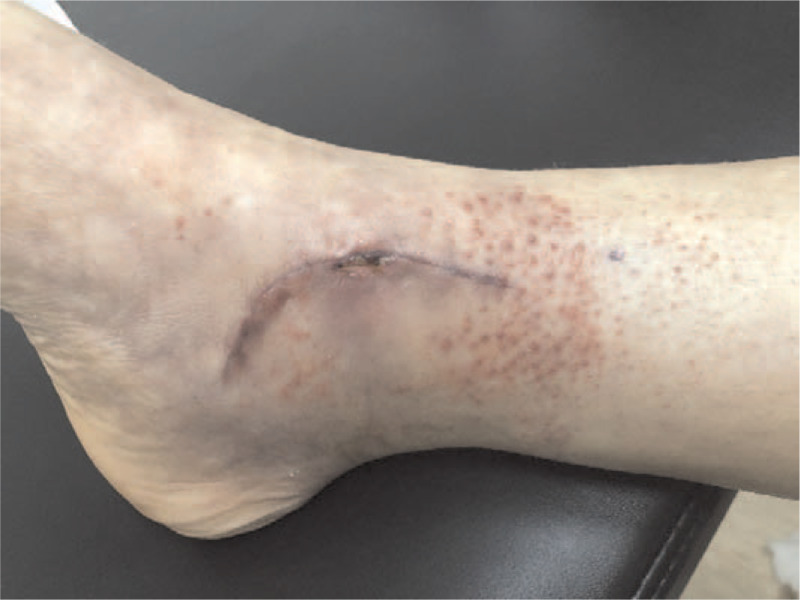
A 37-year-old woman treated for an ankle fracture presented with an itchy erythemato-eczematous and papulovesicular lesion at the surgical wound, 4 weeks postoperatively.

A 37-year-old woman developed an itchy erythemato-eczematous and papulovesicular lesion around her surgical wound on the fourth postoperative week (Fig. 3). After treatment with an antihistamine and local steroid ointment, the skin lesions resolved completely.

#### 3.1.4 Case 4

**Figure 4:**
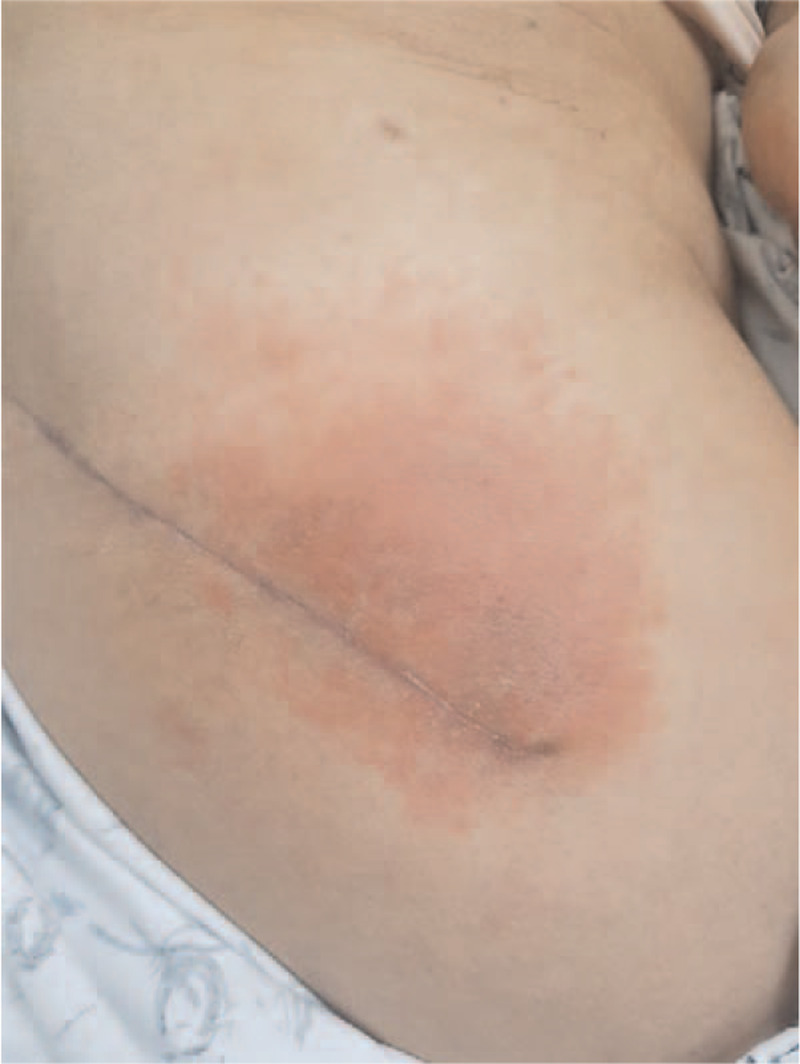
A 62-year-old woman was diagnosed with contact dermatitis characterized by an erythemato-edematous and vesicular lesion around the surgical wound in the seventh postoperative week.

A 62-year-old woman developed an erythemato-edematous and vesicular lesion around her surgical wound on the sixth postoperative week (Fig. 4). She was afebrile, and a routine blood test at the first outpatient clinic follow-up (7 weeks postoperatively) showed: WBC, 4700/μL; ESR, 28 mm/h; and CRP, 0.18 mg/dL. The mesh over her surgical wound had not been removed, and it was removed immediately. We suspected contact dermatitis and referred the patient to the dermatology clinic. After confirming our contact dermatitis diagnosis, local steroid ointment application and oral antihistamines were prescribed, and the patient was cured after 6 weeks of treatment.

## 4 Discussion

In this study of liquid adhesive-related contact dermatitis, 2.8% of patients developed contact dermatitis. Compared with the incidence (29/6008, 0.5%) reported by Chalmers et al,^[11]^ the incidence in our study was higher, whereas it was lower than the incidence reported by Nakagawa et al^[12]^ (7/100, 7%). Chalmers et al^[11]^ used Dermabond Prineo skin closure system (Ethicon Inc., NJ) containing 2-octyl cyanoacrylate and Nakagawa et al^[12]^ used Dermabond Advanced topical skin adhesive (Ethicon Inc., NJ) containing 2-octyl cyanoacrylate, each.

Contact dermatitis may be confused with SSI because an erythemato-edematous lesion appears similar to the redness of SSI, and if contact dermatitis occurs in a few days after surgery, inflammatory markers might be elevated. Generally, contact dermatitis is characterized by erythemato-edematous vesicular or crusted lesions, while, SSI is characterized by a pyrogenic effusion accompanied by a foul odor, bleeding from the wound, pain, and an abscess.^[13]^Table 2 summarizes the clinical manifestations of contact dermatitis and SSIs. Based on these clinical features, allergic contact dermatitis could be differentiated from SSI, and a comprehensive assessment should be performed, including a careful wound examination, blood tests, in collaboration with dermatologists.

**Table 2:** Summary of clinical features of contact dermatitis and surgical site infection.

Contact dermatitis	Surgical site infection
Pruritic, burning, stinging pain	New or increased pain
Erythema	Erythema
Oedema, swelling	Oedema, swelling
Dry skin	Purulent discharge
Blistering	Abscess
Vesicles	Malodor
Delayed healing	Delayed healing
Crusting	Wound bed discoloration
Pruritis, eczema	Bleeding, friable wound
Periwound weeping	Increased wound exudate

If treatment is delayed due to the delayed diagnosis, secondary complications, such as infection, could occur due to the penetration of the fragile skin by bacteria.^[10]^ Conversely, prompt diagnosis and treatment may improve the prognosis of contact dermatitis. When contact dermatitis is clinically suspected after the use of the liquid adhesive system, the mesh should be removed immediately. Surgeons should inform patients of the possibility of postoperative wound complications before a skin adhesive is utilized. Furthermore, the wound should be monitored carefully after the use of skin adhesives to avoid a delay in the diagnosis and treatment of contact dermatitis.

Skin adhesive-related contact dermatitis results from a type IV hypersensitivity reaction, which is a delayed reaction to a substance to which the patient was previously exposed.^[12]^ It often presents with pruritis, eczema, oedema, and blisters, usually beginning 1 to 2 weeks after exposure. Allergic contact dermatitis is diagnosed by identifying a typical combination of skin lesion, previous exposure to the antigen, consistent patch and blood test results and tissue examination findings, and appropriate response to the dermatitis treatment. Although patch tests were not used for the diagnosis of allergic contact dermatitis in this study, patch tests, by 2-hydroxyethyl methacrylate (2-HEMA) or acrylate itself, could be used to detect the hypersensitivity to (meth)acrylates. Furthermore, the allergological tests results in patients with suspicious exposure to acrylates before surgery can be an important factor for deciding whether to use a liquid skin adhesive for the skin closure or not.^[14,15]^

The risk factors for contact dermatitis include high-risk occupations that result in exposure to antigens (e.g., medical personnel, chemical plant and construction workers, beauticians, and mechanics), advanced age, and a history of atopic dermatitis.^[16–21]^ There are reports about allergic contact dermatitis in non-occupational settings after exposure to acrylates or methacrylates in gel nail polish, electrodes of electrocardiogram, and dental prosthesis.^[22–26]^ Furthermore, a report indicated that low humidity could also be a risk factor after the use of a skin adhesive, because aridity can affect cyanoacrylate polymerization.^[27]^ The liquid skin adhesive used in our study was Dermabond Prineo system containing 2-octyl cyanoacrylate. Due to its cross-reactivity, allergic contact dermatitis can occur after appliance of the Dermabond Prineo system for skin closure, following previous sensitization to acrylates or methacrylates, and the number of allergic contact dermatitis cases after using Dermabond Prineo system is increasing.^[28,29]^ In their analysis of 29 individuals who had skin adhesive-related contact dermatitis, Chalmers et al^[11]^ found that 8 (28%) had previous surgical exposure to the skin adhesive, and 7 (24%) had suspected exposure as medical workers or while undergoing surgery. In our study, 1 patient was a doctor, and we could not rule out previous exposure to skin adhesives. Skin adhesives should be used cautiously in patients with a history of surgical skin adhesive application and in medical personnel, given their high risk of contact dermatitis due to previous exposure.

We identified no other complications, such as infection, dehiscence, or the need for further wound closure. Similarly, studies conducted by Chalmers et al^[11]^ did not identify any complications related to the wound healing, such as infection or dehiscence. Krishnamoorthy et al^[30]^ used Dermabond (Ethicon UK, Edinburgh, UK) containing 2-octyl cyanoacrylate, and there was no significant difference in the rate of hematoma occurrence between the use of skin adhesives and nylon suture. In our study, there was no hematoma occurrence, and a drain was placed after arthroplasty surgeries to prevent hematomas because blood and other fluids cannot flow from the wound after it is closed with a skin adhesive.

Our study had several limitations. We included a relatively small number of patients. Thus, our findings may not represent the overall incidence of contact dermatitis due to skin adhesives. Moreover, an allergological workup, which is crucial for an etiological diagnosis of allergic contact dermatitis, was totally absent in our study. Additionally, the retrospective design did not permit us to investigate patient satisfaction or economic effects, and we focused only on complications caused by skin adhesives.

In conclusion, the surgical wound healed in all cases where a skin adhesive was used. However, contact dermatitis occurred in 2.8% of our patients. Therefore, clinicians should be aware of the risk of skin adhesive-related contact dermatitis and monitor the surgical wound regularly in cases where they are used.

## Author contributions

**Conceptualization:** Ji Wan Kim.

**Data curation:** Sang Pil So.

**Formal analysis:** Sang Pil So.

**Funding acquisition:** Ji Wan Kim.

**Investigation:** Jae Youn Yoon.

**Methodology:** Ji Wan Kim.

**Project administration:** Jae Youn Yoon, Ji Wan Kim.

**Resources:** Ji Wan Kim.

**Software:** Ji Wan Kim.

**Supervision:** Ji Wan Kim.

**Validation:** Jae Youn Yoon, Ji Wan Kim.

**Visualization:** Jae Youn Yoon.

**Writing – original draft:** Sang Pil So.

**Writing – review & editing:** Ji Wan Kim.
